# Clinical Outcomes and Safety Profile of Tranexamic Acid Use in Critically Ill Patients with Bleeding: A Single-Center Retrospective Descriptive Study

**DOI:** 10.3390/healthcare14091178

**Published:** 2026-04-28

**Authors:** Bayader Kalkatawi, Bashaer Saber, Raghad Alhuthil, Hanadi Alahdali, Razan Al-Alkami, Walaa Alsanoosi, Hassan Hawa, Mohammad S. Dairi, Namareq Fahad Aldardeer

**Affiliations:** 1 King Abdulaziz Medical City, Ministry of National Guard-Health Affairs, Jeddah 22384, Saudi Arabia; 2 King Faisal Specialist Hospital and Research Centre, Jeddah 21499, Saudi Arabiah.a.alahdali@gmail.com (H.A.);; 3 Department of Pediatrics, King Faisal Specialist Hospital and Research Centre, Riyadh 12713, Saudi Arabia; 4 Department of Medicine, College of Medicine, Umm Al-Qura University, Makkah 24381, Saudi Arabia

**Keywords:** tranexamic acid, critical care, thrombosis, bleeding

## Abstract

**Highlights:**

**What are the main findings?**

- Tranexamic acid (TXA) was associated with a low thrombotic event rate in critically ill ICU patients, yet 24.8% experienced rebleeding within 30 days, raising concerns about its sustained hemostatic efficacy.
- The APACHE II score was the only significant predictor of 30-day mortality, with an overall mortality rate of 55.8%, reflecting the high disease severity in this population.

**What are the implications of the main findings?**

- The combination of a low thrombotic event rate with a 24.8% rebleeding rate indicates that while TXA appears safe, its sustained hemostatic effectiveness is uncertain, supporting the need for optimized dosing strategies and further prospective studies to refine its role in ICU bleeding management.
- The identification of **APACHE II score** as the sole predictor of 30-day mortality suggests that TXA use alone does not influence survival, emphasizing the need to prioritize early risk stratification and severity-driven management in critically ill bleeding patients.

**Abstract:**

**Background**: Tranexamic acid (TXA) is an antifibrinolytic agent that helps prevent and manage bleeding events. In patients with trauma, TXA reduced bleeding-related deaths by one-third. Moreover, TXA showed a lower risk of mortality from bleeding in postpartum patients. Few studies have examined the appropriateness of TXA use for the management of bleeding in critically ill patients. This study aimed to describe the clinical outcomes and the safety of TXA use in critically ill patients with bleeding. **Methods**: This single-center, single-arm descriptive study was conducted at King Faisal Specialist Hospital & Research Center, Jeddah, between January 2018 and March 2023. The study included adult patients 18 years or older who were admitted to the medical intensive care unit (ICU) for ≥48 h and had documented bleeding that was treated with TXA for at least one dose. The primary outcome was the frequency of thrombotic events. Secondary outcomes included time from bleeding onset to bleeding resolution, rebleeding event at 30 days, time from bleeding onset to rebleeding event, ICU and hospital length of stay, and 30-day all-cause mortality. **Results**: A total of 129 patients were included in the study, 55% of whom were male. The median age was 60.9 years. The median APACHE II score was 22 (15–29). At baseline, 24.8% of patients had a history of bleeding. Major bleeding occurred in 86.1% of the patients. The frequency of thrombotic events was 2.3%. The median bleeding duration was 3.9 days (1.9–7.0). Rebleeding events at 30 days occurred in 24.8% of patients, with a median time of 11.7 days (8–14.8) from bleeding onset to rebleeding. The average ICU length was 12 days (6–24), and the average hospital length of stay was 25 days (15–50). The 30-day all-cause mortality rate was 55.8%. Multivariable analysis assessing factors contributing to mortality revealed that higher APACHE II score was strongly associated with increased mortality (adjusted OR 1.14 per point increase, 95% CI 1.07–1.21, *p* < 0.001), while higher platelet counts were independently protective, with each 10 × 10^9^/L increase associated with a 4% reduction in mortality odds (adjusted OR 0.96, 95% CI 0.93–0.99, *p* = 0.034). **Conclusions:** In this descriptive study, TXA use in critically ill patients was accompanied by low absolute rates of thrombotic and rebleeding events. Further studies with larger sample sizes and comparable groups are needed to examine the appropriateness of TXA use in managing bleeding events in the ICU.

## 1. Background

Bleeding is a medical emergency that requires immediate management. Among intensive care unit (ICU) patients, bleeding was reported in up to 50%; patients admitted to the ICU regularly experience bleeding for several etiologies linked to higher morbidity and mortality rates [1]. The potential etiology of bleeding is divided into inherited bleeding disorders, including inherited platelet disorders, congenital hemophilia, and von Willebrand disease. Acquired bleeding includes acquired thrombocytopenia, acquired abnormalities of platelet function, reduced production of coagulation factors, massive transfusion, acquired hemophilia, acquired von Willebrand syndrome, and invasive procedures such as inserting central venous catheters [2,3,4,5,6,7,8]. Due to the complexity of the underlying causes, managing bleeding in ICU patients remains challenging.

Tranexamic acid (TXA) is an antifibrinolytic agent essential in bleeding management. TXA functions by forming a reversible complex that displaces plasminogen from fibrin, inhibits fibrinolysis, and prevents plasmin from acting as a proteolytic enzyme. TXA is FDA-approved for short-term use for these two indications: chronic abnormal uterine bleeding and tooth extraction in patients with hemostatic defects. At the same time, it has been widely used for off-label indications, such as trauma-associated hemorrhage or traumatic brain injury, postpartum hemorrhage treatment or prevention, perioperative prevention of blood loss and transfusion, and intracranial hemorrhage associated with thrombolytic therapy.

TXA has demonstrated benefit across multiple bleeding contexts, particularly in trauma and postpartum hemorrhage, where early administration reduces bleeding-related mortality without increasing thrombotic risk [9,10]. Evidence from other settings, including gastrointestinal bleeding and hemoptysis, suggests improvements in bleeding control and resource utilization [11,12], although randomized trials such as HALT-IT and STOP-AUST have shown no significant differences in mortality or thromboembolic outcomes compared to placebo [13,14].

However, critically ill medical ICU patients represent a distinct and understudied population with different bleeding mechanisms, comorbidities, and risk profiles. Accordingly, this study aimed to describe the clinical outcomes and safety of TXA use in critically ill patients with bleeding. We hypothesized that TXA use would be associated with a low observed frequency of thrombotic events, the primary endpoint of this study.

## 2. Methodology

### 2.1. Study Design

Single-center, single-arm, retrospective descriptive study. The study was conducted in the medical ICU at the tertiary care hospital King Faisal Specialist Hospital & Research Centre, Jeddah, Saudi Arabia, between 1 January 2018 and 30 March 2023; the hospital’s Institutional Review Board approved the study (IRB 2023-65) on 17 September 2023. This study was conducted and reported in accordance with the STROBE (Strengthening the Reporting of Observational Studies in Epidemiology) guidelines, and the completed checklist is provided as Supplementary Materials.

### 2.2. Patient Population and Data Collection

The study included medical ICU patients aged 18 years and older who were admitted for 48 h or longer, had documented bleeding, and received at least 1 dose of TXA. We excluded patients who were admitted to the surgical ICU, trauma patients, or those who were on TXA before the onset of bleeding. Data were collected using REDCap and included the following: demographics, type of bleeding, comorbidities, reason for ICU admission, site of bleeding, cumulative dose, and duration of TXA.

### 2.3. Study Endpoints

The primary endpoint was the frequency of thrombotic events. Secondary endpoints included the incidence of rebleeding events at 30 days and the time from bleeding onset to bleeding resolution. Additionally, the study assessed the time from TXA initiation to bleeding resolution, the number of blood products received, all-cause mortality at 30 days, the length of ICU stays, and the length of hospital stay.

### 2.4. Definitions

We defined major bleeding according to the International Society of Thrombosis and Hemostasis (ISTH) any fatal bleeding; and/or symptomatic bleeding in a critical area or organ, such as intracranial, intraspinal, intraocular, retroperitoneal, intra-articular or pericardial, or intramuscular with compartment syndrome; and/or bleeding causing a fall in haemoglobin levels of 1.24 mmol/L (20 g/L or greater) or more or leading to a transfusion of ≥2 U of whole blood or red cells. Minor bleeding was defined as any bleeding event that did not meet the ISTH criteria for major bleeding. We defined rebleeding as an additional transfusion requirement and/or a ≥20% decrease in hematocrit after at least 24 h of clinical stability. This definition was adopted as a study-specific operational definition, as no universally accepted definition of rebleeding exists in critically ill populations, and is consistent with established ISTH principles that use hemoglobin decline and transfusion requirement as markers of clinically significant bleeding. Thrombosis is defined as the formation of a blood clot, known as a thrombus, within a blood vessel. It prevents blood from flowing normally through the circulatory system. (deep vein thrombosis, pulmonary embolism, stroke, and myocardial infarction) Based on the ISTH definition. Total blood products were calculated as the cumulative number of transfused units per patient during the study period, including packed red blood cells, platelets, fresh frozen plasma, and other blood components.

### 2.5. Statistical Analysis

This was a single-arm descriptive study without a control group. All eligible patients who met the inclusion criteria during the study period were included. Frequencies and percentages were reported for categorical variables, and medians and interquartile ranges (IQRs) for continuous variables. The Shapiro–Wilk test was used to assess normality. Univariable analysis to investigate 30-day mortality-related risk factors was done using Fisher’s exact test for categorical variables and the Mann–Whitney U test for continuous variables. Additionally, a multivariable logistic regression model was used to adjust for confounders. A complete case analysis was performed, and no imputation methods were applied. Variables were selected based on clinical relevance and those with *p* < 0.10 in univariable analysis. Collinearity was assessed using variance inflation factors (VIF), and model assumptions were evaluated using standard diagnostic approaches. To explore TXA dosing heterogeneity, patients were stratified into two groups based on total dose (≤1000 mg vs. >1000 mg). Baseline characteristics, bleeding features, and outcomes were compared between groups using Fisher’s exact test and the Mann–Whitney U To address potential sources of bias, we applied predefined eligibility criteria to reduce selection bias. Data were collected using standardized definitions and protocols to minimize information bias. Potential confounders were identified a priori and adjusted for using multivariable regression analysis. Analyses were performed using STATA software version 18, and significance was determined at α = 0.05.

## 3. Results

**Figure 1:**
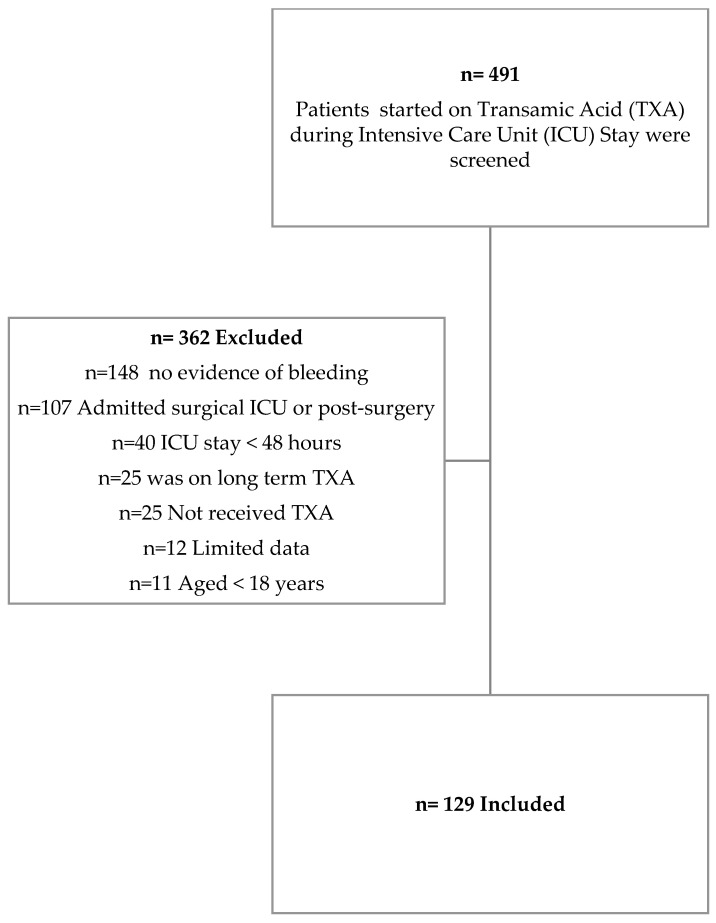
Patient Screening and Selection.

Of the 491 patients screened, the overall study population included 129 critical care patients who received TXA (Figure 1) with a median age of 60.9 years (Table 1). More than half (55.0%) of the patients were male. The median and interquartile range (IQR) for patients’ APACHE II scores were 22.0 (15. 0–29.0). Just over half of the patients were admitted to the ICU for either hemorrhagic shock (28.7%) or respiratory failure (27.1%). Almost one-third of the participants (31.8%) had hypertension, and only over one-fifth (21.7%) had diabetes mellitus (Table 1).

Approximately half (48.1%) of the participants used an anticoagulant at the time of bleeding, and the majority (86.1%) experienced major bleeding during their hospital stay, with over half (54.3%) of the patients having gastrointestinal hemorrhage as the site of bleeding. The median (IQR) TXA dose was 1000 mg (857–1833 mg), administered over a median duration of 2 days (Table 2).

Three patients experienced a thrombotic event within three months of receiving TXA and bleeding onset, with a median (IQR) of 8.3 (7.4–39.1) days (Table 2). Among these three patients, two experienced an event within the first 30 days after starting medication. One-fourth (24.8%) of patients experienced rebleeding, and 62% required blood transfusions, with a median (IQR) of 7.5 (3.4–12.7) units of blood products used. The 30-day all-cause mortality rate was 55.8% (Table 2).

In the univariable analysis, higher illness severity and lower platelet counts were significantly associated with 30-day mortality. Patients who died had a markedly higher median APACHE II score than survivors (25 [IQR: 19–30] vs. 16 [12–23], *p* < 0.001). In addition, baseline platelet counts were significantly lower in non-survivors (111 × 10^9^/L [45–167] vs. 189 × 10^9^/L [112–296], *p* < 0.001). Bleeding during ICU admission was also more frequent among patients who died (59.7% vs. 38.6%, *p* = 0.021). No other variables, including age, sex, BMI, comorbidities, TXA-related variables (dose, timing, or duration), anticoagulation status, or complications such as rebleeding and thrombotic events, were significantly associated with mortality (all *p* > 0.05) (Table 3).

In the multivariable logistic regression model, only illness severity and platelet count remained independent predictors of 30-day mortality. Higher APACHE II score was strongly associated with increased mortality (adjusted OR 1.14 per point increase, 95% CI 1.07–1.21, *p* < 0.001), indicating a 14% increase in the odds of death for each 1-point increase. Conversely, higher platelet counts were independently protective, with each ×10^9^/L increase associated with a 4% reduction in mortality odds (adjusted OR 0.96, 95% CI 0.93–0.99, *p* = 0.034) (Table 4).

Patients receiving higher TXA doses (>1000 mg) had significantly longer bleeding duration compared to the lower dose group (≤1000 mg) (median: 5.3 vs. 3.1 days, *p* = 0.019) and a higher proportion of major bleeding (93.1% vs. 80.3%, *p* = 0.043) (Table 5).

## 4. Discussion

This retrospective descriptive study elucidated the clinical outcomes and safety profile of TXA use in critically ill patients with documented hemorrhages. The findings indicate that TXA administration in this population is associated with a relatively low incidence of thrombotic events (2.3%) and a substantial rate of rebleeding events (24.8%) within 30 days. The absence of a control group limits the ability to attribute observed outcomes directly to TXA use and precludes causal inference; therefore, findings should be interpreted as descriptive and hypothesis-generating.

The observed low rate of thrombotic complications (2.3%) is particularly noteworthy given the elevated risk of thrombosis in critically ill patients. This finding is consistent with previous studies in other clinical contexts. Previous large trials, such as CRASH-2, HALT-IT, and WOMAN, have demonstrated variable effects of TXA across clinical settings, generally supporting its safety profile without a consistent increase in thrombotic events. However, these studies were conducted in trauma, gastrointestinal, and postpartum bleeding populations, respectively, which differ significantly from critically ill ICU patients [8,13,15].

Our results extend these safety observations to the critical care setting, suggesting that the thrombotic risk associated with TXA administration may be acceptable, even in this high-risk population. The absence of significant associations between various comorbidities and thrombotic events in our univariate analysis was noteworthy and unanticipated. This observation may be attributed to the limited number of thrombotic events in our cohort (n = 3), which may have limited the statistical power to detect such associations. Additionally, this may suggest that conventional risk factors for thrombosis may be overshadowed by the overall severity of illness and other acute factors within the complex context of critical illness.

The rebleeding rate of 24.8% observed in this study is clinically notable and reflects the complexity of bleeding in critically ill patients. As this is a descriptive cohort without a comparator, this finding should be interpreted as an observed event rate rather than an indicator of treatment effectiveness. It suggests that, while TXA may contribute to initial bleeding control, its sustained hemostatic effect in critically ill patients may be limited. This finding likely reflects the complex and multifactorial nature of bleeding in this population, where underlying disease severity, comorbidities, and ongoing physiological derangements may contribute to recurrent bleeding despite antifibrinolytic therapy. This rate exceeds that observed in some studies of TXA in alternative contexts. For instance, in the CRASH-2 trial, the necessity for additional blood products (a proxy for ongoing or recurrent bleeding) was significantly reduced in the TXA group (RR 0.98, 95% CI 0.96–0.99, *p* = 0.004) [8]. In the context of postpartum hemorrhage, the WOMAN trial reported a lower rate of additional uterotonic use (a marker of ongoing bleeding) in the TXA group than in the placebo group (33.6% vs. 35.3%, *p* = 0.014) [15]. The elevated rebleeding rate in our study potentially reflects the complex and often multifactorial nature of bleeding in critically ill patients, emphasizing the challenges in achieving sustained hemostasis in this population.

This finding highlights an important source of clinical heterogeneity in our study. TXA dosing in this cohort appeared to be individualized rather than protocol-driven, likely reflecting differences in bleeding severity, anatomical site, and clinician judgment. As a result, higher doses were more frequently administered in patients with more severe or prolonged bleeding. This confounding by indication limits the ability to interpret dose-outcome relationships and suggests that observed associations between higher dose and worse outcomes are unlikely to be causal. Instead, TXA dose should be viewed as a marker of disease severity rather than an independent determinant of clinical outcomes.

The median time to bleeding resolution observed in this study (3.9 days) provides valuable insights into the potential efficacy of TXA in this patient population. While there is limited comparative data on bleeding resolution time in critically ill patients, this finding suggests that TXA may contribute to relatively rapid hemostasis in many cases. A study by Wand et al. on inhaled TXA for hemoptysis reported a median time to cessation of bleeding of 2 days in the TXA group versus 4 days in the placebo group [12]. However, the wide IQR in our study (1.9–7.0 days) underscores the variability in response, likely reflecting the heterogeneity of bleeding etiologies and severities encountered in the critical care setting.

The 30-day all-cause mortality rate of 55.8% observed in our cohort is notably high and likely reflects the severity of illness in this population rather than the effect of TXA itself. This rate exceeds that reported in some studies of critically ill patients with bleeding, such as the HALT-IT trial of TXA in gastrointestinal bleeding (9.5% mortality at 28 days) [13] Higher illness severity and thrombocytopenia emerged as the only independent predictors of 30-day mortality in this cohort. Specifically, each 1-point increase in the APACHE II score was associated with a 14% increase in the odds of death, highlighting the dominant impact of baseline physiological derangement and critical illness burden on outcomes. In parallel, lower platelet counts were independently associated with increased mortality, likely reflecting both the severity of bleeding and underlying coagulopathy or bone marrow dysfunction in this critically ill population. Although bleeding during ICU admission was associated with mortality in univariable analysis, this effect was not sustained after adjustment, suggesting that it may be mediated by overall illness severity rather than representing an independent risk factor. Importantly, TXA-related variables, including cumulative dose and timing of administration, were not associated with mortality, supporting the notion that outcomes are primarily driven. This finding is consistent with the established role of the APACHE II as a predictor of mortality in critically ill patients in many studies [16,17].

Our study had several limitations that merit consideration. This retrospective, single-center design constrains the generalizability of our findings and precludes causal inferences. The absence of a control group makes it challenging to attribute the outcomes to TXA use. Furthermore, no formal prior sample size calculation was performed because this was a retrospective descriptive study that included all eligible patients during the study period. However, because thrombotic events were rare (3/129, 2.3%), the study had limited precision for estimating this outcome and limited statistical power to detect associations with rare events. Notwithstanding these limitations, the present study provides significant real-world data on the utilization of TXA in critically ill patients with bleeding. This population has been underrepresented in previous studies of TXA. These findings suggest that TXA use in this cohort was associated with a low observed incidence of thrombotic events. However, the substantial rebleed rate underscores the necessity for meticulous monitoring and potentially adjunctive therapies in these patients. Importantly, this study was limited to a medical ICU population, which reduced clinical heterogeneity and allowed for a more focused evaluation of TXA use in medically complex critically ill patients; however, it limits the generalizability of our findings. Therefore, the findings should not be extrapolated to trauma or perioperative patients, where TXA use, timing, and bleeding mechanisms differ substantially.

Future research should encompass prospective, multicenter studies with larger sample sizes to validate these findings. Randomized controlled trials comparing TXA to placebo or standard care in critically ill patients with bleeding would provide more robust evidence of efficacy and safety. Moreover, investigations into optimal dosing regimens, timing of TXA administration, and identification of patient subgroups most likely to benefit from TXA therapy would be valuable for refining clinical practice guidelines for this challenging patient population.

## 5. Conclusions

In this descriptive cohort of critically ill patients with bleeding, TXA use was associated with low observed rates of thrombotic events and a clinically notable rate of rebleeding. Given the absence of a comparator group and the study design, these findings should be interpreted as descriptive and hypothesis-generating rather than indicative of comparative efficacy or safety. Moreover, the notable high mortality rate may highlight the complexity of this population and suggests that outcomes are likely driven by underlying disease severity rather than the intervention alone. Given the absence of a comparator group and the limited sample size, these findings should be interpreted cautiously and considered hypothesis-generating. Further prospective, multicenter randomized controlled trials are needed to better define the safety and efficacy of TXA in critically ill patients.

## Figures and Tables

**Table 1:** Baseline Characteristics of Critical Care Patients Who Received Tranexamic Acid (n = 129).

Characteristic	Median (IQR) OR n (%)
Age, years, median (IQR)	60.9 (45.1–72.8)
Gender, n (%)	
Female	58 (45.0)
Male	71 (55.0)
BMI, kg/m^2^, median (IQR)	27.1 (23.1–32.1)
APACHE II Score, median (IQR) (n = 124)	22 (15–29)
APACHE II Mortality Probability, %, median (IQR) (n = 124)	40 (25–55)
Reason for ICU admission, n (%)	
Sepsis/septic shock	30 (23.3)
Hemorrhagic shock	37 (28.7)
Respiratory failure	35 (27.1)
Decreased LOC	21 (16.3)
GI bleeding	9 (7.0)
Comorbidities, n (%)	
Diabetes	28 (21.7)
Hypertension	41 (31.8)
Atrial fibrillation	10 (7.8)
Chronic kidney disease	15 (11.6)
End-stage renal disease	7 (5.4)
Liver disease	9 (7.0)
Patient history, n (%)	
History of ischemic stroke	13 (10.1)
History of bleeding (any site)	32 (24.8)

IQR = interquartile range; LOC = level of consciousness; GI = gastrointestinal.

**Table 2:** Bleeding Characteristics, Management and Clinical Outcomes (n = 129).

Characteristic	Median (IQR) OR n (%)
Bleeding event location, n (%)	
During ICU admission	65 (50.4)
Before ICU admission	64 (49.6)
Type of bleeding, n (%)	
Minor bleeding	18 (14.0)
Major bleeding	111 (86.1)
Site of bleeding, n (%)	
Intracranial hemorrhage	9 (7.0)
Gastrointestinal hemorrhage	70 (54.3)
Hematuria	4 (3.1)
Epistaxis	4 (3.1)
Rectal bleeding	11 (8.5)
Therapeutic anticoagulation at bleeding onset, n (%)	62 (48.1)
DVT prophylaxis at bleeding onset, n (%)	21/67 (31.3)
Blood product transfusion received, n (%)	
RBC transfusion	80 (62.0)
FFP transfusion	76 (58.9)
PCC transfusion	100 (77.5)
Platelet transfusion	66 (51.2)
Vitamin K	68 (52.7)
Total RBC units transfused, median (IQR)	7.5 (3.4–12.7)
TXA total dose, mg, median (IQR)	1000 (857–1833)
TXA duration, days, median (IQR)	2 (1–5)
Time from TXA start to bleeding stop, days, median (IQR)	3 (1–6)
Total bleeding duration, days, median (IQR)	3.9 (1.9–7.0)
New seizures after TXA, n (%)	25 (19.4)
Thrombotic events within 30 days, n (%)	2 (1.6)
Thrombotic events within 90 days, n (%)	3 (2.3)
PE	1 (0.8)
DVT	1 (0.8)
Ischemic stroke	1 (0.8)
Time from bleeding onset to thrombotic event (days)	8.3 (7.4–39.1)
Rebleeding, n (%)	32 (24.8)
Time from bleeding onset to rebleeding event (days)	11.7 (8.0–14.8)
Length of ICU stay, days, median (IQR)	12 (6–24)
Length of hospital stay, days, median (IQR)	25 (15–50)
All-cause mortality at 30 days, n (%)	72 (55.8)

DVT = deep vein thrombosis; RBC = red blood cell; FFP = fresh frozen plasma; PCC = prothrombin complex concentrate; TXA = tranexamic acid; PE = pulmonary embolism; ICU = intensive care unit.

**Table 3:** Univariable Analysis of Factors Associated with 30 Day Mortality (n = 129).

Variable	Died (n = 72)	Alive (n = 57)	*p*-Value
Demographics			
Age, years, median (IQR)	63.2 (43.4–72.6)	59.2 (45.7–73.1)	0.812 ^a^
Male sex, n (%)	41 (56.9)	30 (52.6)	0.722 ^b^
BMI, kg/m^2^, median (IQR)	26.5 (23.0–31.3)	28.7 (23.7–33.5)	0.294 ^a^
Severity scores			
APACHE II Score, median (IQR) (n = 124)	25 (19–30)	16 (12–23)	**<0.001** ^a^
ICU admission reason, n (%)			
Sepsis/septic shock	21 (29.2)	9 (15.8)	0.094 ^b^
Hemorrhagic shock	16 (22.2)	21 (36.8)	0.080 ^b^
Respiratory failure	19 (26.4)	16 (28.1)	0.845 ^b^
Decreased LOC	15 (20.8)	6 (10.5)	0.151 ^b^
GI bleeding	3 (4.2)	6 (10.5)	0.182 ^b^
Comorbidities, n (%)			
Hypertension	27 (37.5)	14 (24.6)	0.131 ^b^
Diabetes mellitus	18 (25.0)	10 (17.5)	0.391 ^b^
Atrial fibrillation	7 (9.7)	3 (5.3)	0.511 ^b^
CKD	11 (15.3)	4 (7.0)	0.175 ^b^
Leukemia	4 (5.6)	0 (0.0)	0.129 ^b^
Bleeding characteristics			
Bleeding during ICU admission (vs. prior), n (%)	43 (59.7)	22 (38.6)	**0.021** ^b^
Major bleeding (vs. minor), n (%)	62 (86.1)	49 (86.0)	1.000 ^b^
Intra-abdominal hemorrhage, n (%)	5 (6.9)	0 (0.0)	0.066 ^b^
Epistaxis, n (%)	4 (5.6)	0 (0.0)	0.129 ^b^
Bleeding duration (days)	4 (2.3–8.7)	3.8 (1.8–5.9)	0.136 ^a^
Laboratory values			
Baseline Hb, g/L, median (IQR) (n = 120)	80.5 (73.5–91)	83.5 (76–101.5)	0.085 ^a^
Hb 1 day after bleeding, g/L, median (IQR) (n = 127)	78 (67–88)	83 (71–93)	0.087 ^a^
Baseline platelets, ×10^9^/L, median (IQR) (n = 120)	111 (45–167)	189 (112–296)	**<0.001** ^a^
Platelets at bleeding day, ×10^9^/L, median (IQR) (n = 16)	82 (44–215)	262 (133–301)	0.065 ^a^
D-dimer, mg/L, median (IQR) (n = 44)	4.3 (2.6–18.7)	3.9 (1.6–7.1)	0.309 ^a^
TXA exposure			
Time from bleeding to TXA start (days)	0.5 (0.5–1)	0.5 (0.5–1)	0.690 ^a^
TXA total dose (mg), median (IQR)	1000 (986–1719)	1000 (744–1857)	0.579 ^a^
TXA duration (days), median (IQR)	1.5 (1–6)	2 (1–4)	0.611 ^a^
Anticoagulation			
Therapeutic anticoagulation for bleeding, n (%)	32 (44.4)	30 (52.6)	0.380 ^b^
DVT prophylaxis at bleeding, n (%) *	13 (32.5)	8 (29.6)	1.000 ^b^
Complications/adverse events			
New seizures after TXA, n (%)	16 (22.2)	9 (15.8)	0.381 ^b^
Thrombotic event within 90 days, n (%)	3 (4.2)	0 (0.0)	0.254 ^b^
Rebleeding, n (%)	21 (29.2)	11 (19.3)	0.223 ^b^

APACHE II = Acute Physiology and Chronic Health Evaluation II; BMI = Body mass index; DVT = Deep vein thrombosis; GI = Gastrointestinal; Hb = Hemoglobin; ICU = Intensive care unit; IQR = Interquartile range; LOC = Level of consciousness; TXA = Tranexamic acid. ^a^ Mann–Whitney U test (two-tailed); ^b^ Fisher’s exact test (two-tailed). * Denominator for DVT prophylaxis includes only patients not on therapeutic anticoagulation at the time of bleeding (n = 67: 27 alive, 40 dead). Bold *p*-values indicate statistical significance at the *p* < 0.05 level.

**Table 4:** Multivariable Logistic Regression for Factors Associated with 30 Day Mortality (n = 114).

Variable *	Adjusted OR	95% CI	*p*-Value
Male gender (vs. female)	1.05	0.41–2.66	0.922
Age (per year)	0.98	0.96–1.01	0.215
BMI (per kg/m^2^)	0.95	0.89–1.01	0.108
APACHE II score	1.14	1.07–1.21	**<0.001**
Bleeding during ICU admission (vs prior)	1.29	0.46–3.58	0.630
Platelets (per ×10^9^/L increase)	0.96	0.93–0.99	**0.034**
TXA total dose (per 1000 mg increase)	1.26	0.64–2.44	0.505
Duration from TXA start to bleeding stop (per day)	1.03	0.93–1.14	0.556

OR: Odds ratio; CI = Confidence interval; APACHE II = Acute Physiology and Chronic Health Evaluation II; BMI = Body mass index; TXA = Tranexamic acid. * Variables were selected for multivariable modeling based on clinical relevance and those with *p* < 0.10 in univariable analysis. Bold *p*-values indicate statistical significance at the *p* < 0.05 level.

**Table 5:** Comparison of Patient and Bleeding Characteristics by TXA Dose Category (≤1000 mg vs. >1000 mg).

Characteristic	>1000 mg (n = 58)	≤1000 mg (n = 71)	*p*-Value
Demographics and severity			
Age, years, median (IQR)	55.9 (41.1–71.4)	64.0 (45.7–73.0)	0.478 ^a^
APACHE II score, median (IQR) (n = 124)	23 (15–29)	20 (15–29)	0.928 ^a^
Platelets at bleeding day, ×10^9^/L, median (IQR) (n = 16)	113 (43–290)	232 (100–266)	0.266 ^a^
Bleeding characteristics			
Major bleeding (vs. minor), n (%)	54 (93.1)	57 (80.3)	0.043 ^b^
Bleeding duration, days, median (IQR)	5.3 (2.5–8.9)	3.1 (1.8–5.5)	0.019 ^a^
Therapeutic anticoagulation at bleeding onset, n (%)	28 (48.3)	34 (47.9)	1.000 ^b^
Bleeding site, n (%)			
Intracranial hemorrhage	7 (12.1)	2 (2.8)	0.077 ^b^
Gastrointestinal hemorrhage	30 (51.7)	40 (56.3)	0.723 ^b^
Epistaxis	1 (1.7)	3 (4.2)	0.627 ^b^
Rectal bleeding	6 (10.3)	5 (7.0)	0.541 ^b^
Intra-abdominal hemorrhage	2 (3.4)	3 (4.2)	1.000 ^b^
Vaginal hemorrhage	3 (5.2)	2 (2.8)	0.657 ^b^
Pulmonary hemorrhage	1 (1.7)	3 (4.2)	0.627 ^b^
Tracheostomy site bleeding	1 (1.7)	4 (5.6)	0.378 ^b^

^a^ Mann–Whitney U test (two-tailed); ^b^ Fisher’s exact test (two-tailed).

## Data Availability

The data presented in this study are available from the corresponding author upon request due to privacy/ethical restrictions.

## Supplementary Materials

The following supporting information can be downloaded at: https://www.mdpi.com/article/10.3390/healthcare14091178/s1, Table S1: STROBE Statement—checklist of items that should be included in reports of observational studies.

## Abbreviations

**Abbreviation**	**Full Form**
AF	Atrial Fibrillation
APACHE II	Acute Physiology and Chronic Health Evaluation II
BMI	Body Mass Index
BMT	Bone Marrow Transplant
CI	Confidence interval
CKD	Chronic Kidney Disease
COPD	Chronic Obstructive Pulmonary Disease
DM	Diabetes Mellitus
DVT	Deep Vein Thrombosis
HIV	Human Immunodeficiency Virus
HTN	Hypertension
ISTH	International Society of Thrombosis and Hemostasis
ICU	Medical intensive care unit
IQR	Interquartile range
IRB	Institutional Review Board
LOC	Level of Consciousness
MI	Myocardial Infarction
OR	Odds ratio
RCT	Randomized controlled trial
RR	Relative risk
SD	Standard deviation
VIF	Variance inflation factors
WHO	World Health Organization
TXA	Tranexamic acid
